# Supplementary material for: Drug-Tolerant Mycobacterium tuberculosis Adopt Different Survival Strategies in Alveolar Macrophages of Patients with Pulmonary Tuberculosis
Source: Int J Mol Sci. 2023 Oct 6;24(19):14942. doi: 10.3390/ijms241914942 (PMC10573496; doi:10.3390/ijms241914942)
Supplement: Supplementary file 1 [file ijms-24-14942-s001.zip › ijms-2587999-supplementary.pdf]

Supplementary Materials

Figure S1

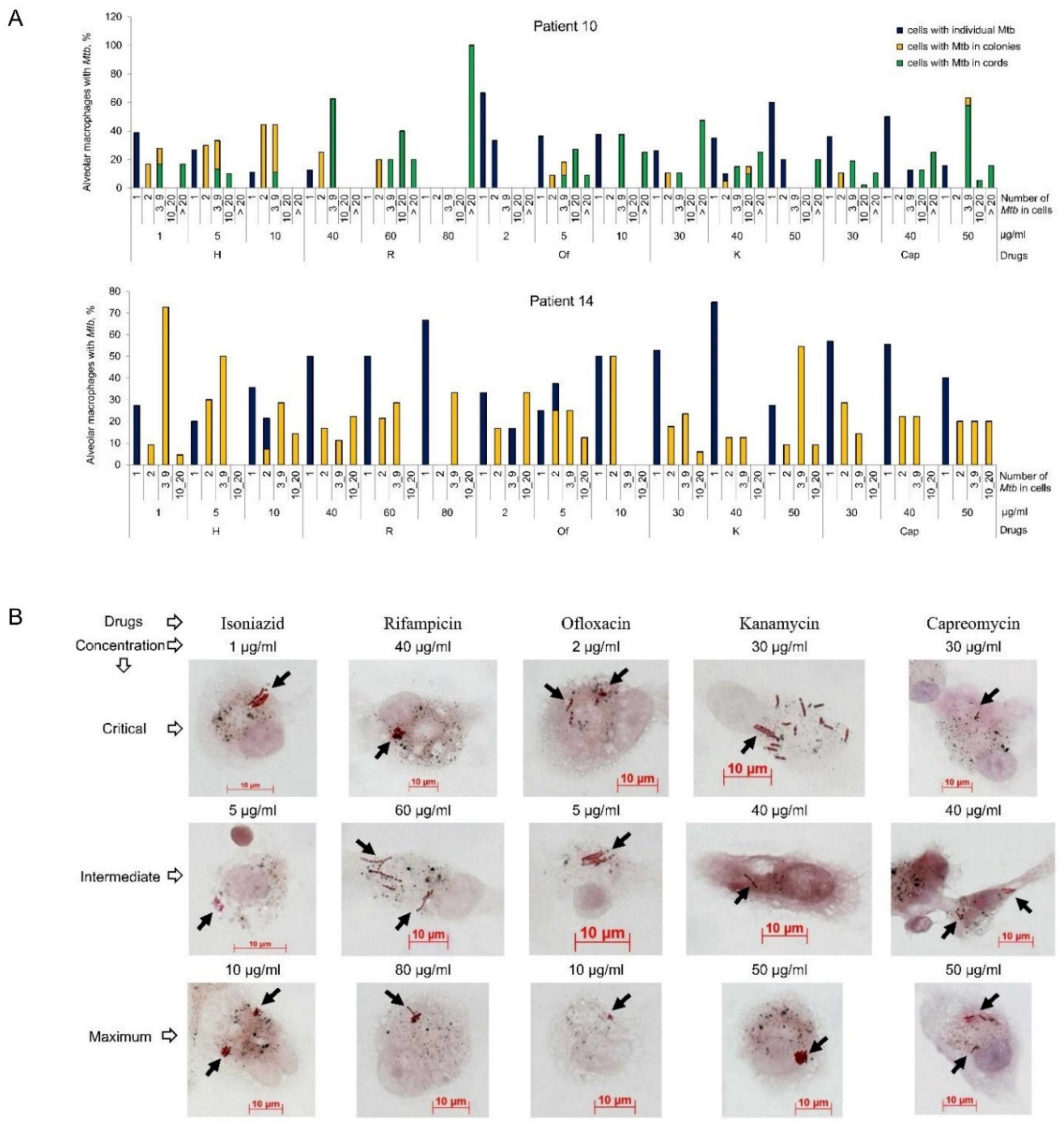

Figure S1. *M. tuberculosis* (*Mtb*) are able to survive exposure to different concentrations of anti-TB drugs in the *ex vivo* cultures of alveolar macrophages obtained from the distant lung tissues of patient 10 with XDR-TB and patient 14 with isoniazid-resistant TB. (**a**) The number of alveolar macrophages with a particular number of acid-fast *Mtb* in them expressed as the percentage of the total number of infected alveolar macrophages does not change significantly between the *ex vivo* cell cultures exposed to different concentrations of anti-TB drugs during three days. Drugs: H, isoniazid; R, rifampicin; Of, ofloxacin; K, kanamycin; Cap, capreomycin. (**b**) The colonies of acid-fast *Mtb* stained by the ZN method and indicated by black arrows are demonstrated on representative images of viable alveolar macrophages without apoptotic or necrotic morphology exposed to different concentrations of anti-TB drugs in *ex vivo* culture during three days for patient 14. The scale bars are 10 µm each.

Figure S2

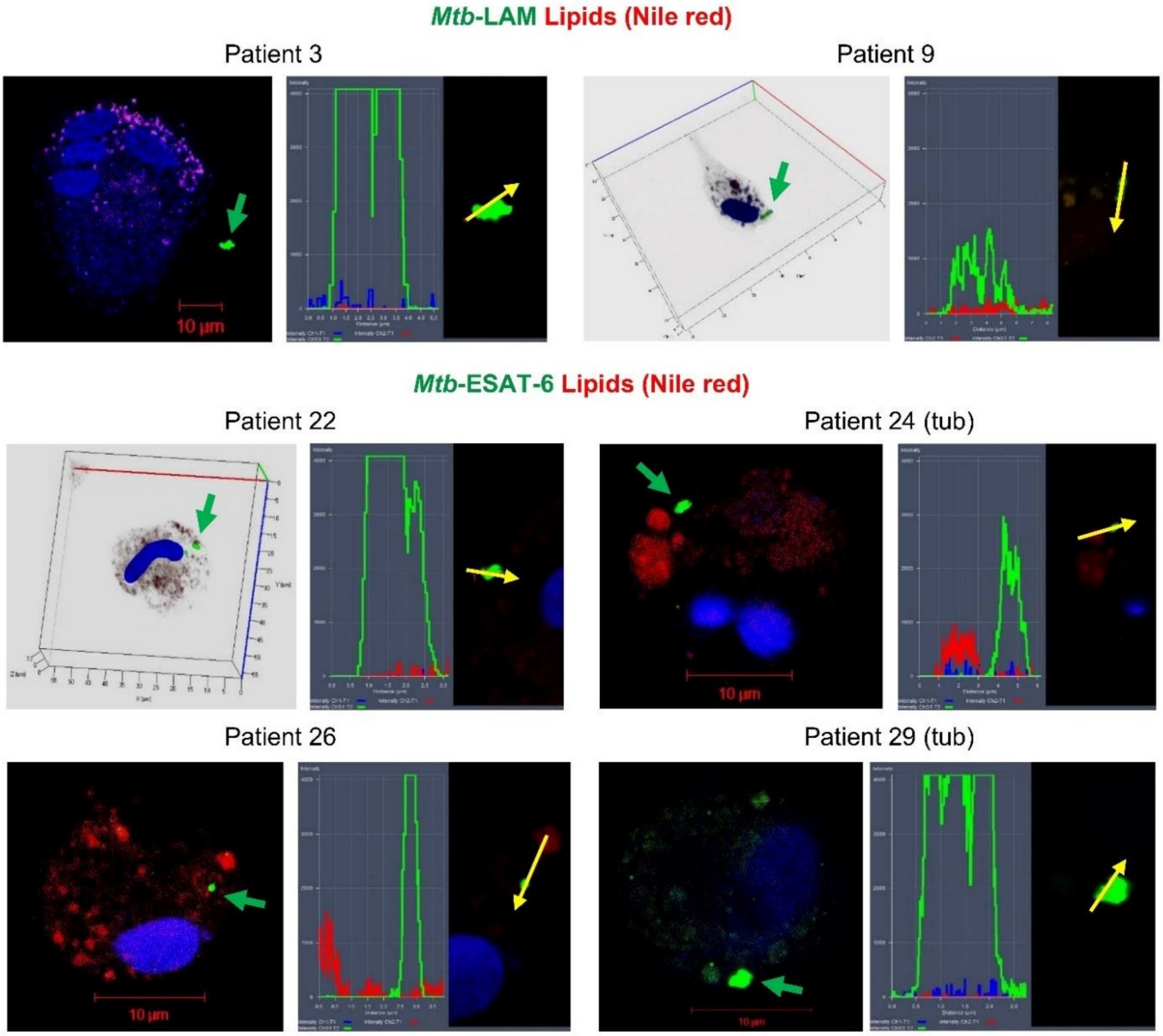

Figure S2. Intracellular lipophilic inclusions are not found in the *Mtb* located both within and outside the alveolar macrophages obtained from the tuberculoma walls (tub) and the tissues distant from the macro-TB lesions of the patients' resected lung parts on representative confocal 3D or single immunofluorescent images stained by the *Mtb* LAM- or ESAT-6-specific antibodies (green signal) and Nile red dye (red signal) after *ex vivo* culture for 16-18 hours. Nuclei are stained by DAPI (blue signal). Green arrows point to *Mtb* (solitary or as colonies). To the right of these images: the profile images of the arrow-marked *Mtb* shown. Yellow arrows point to the areas for constructing profile graphs. The scale bars are 10 µm each.

Figure S3

A

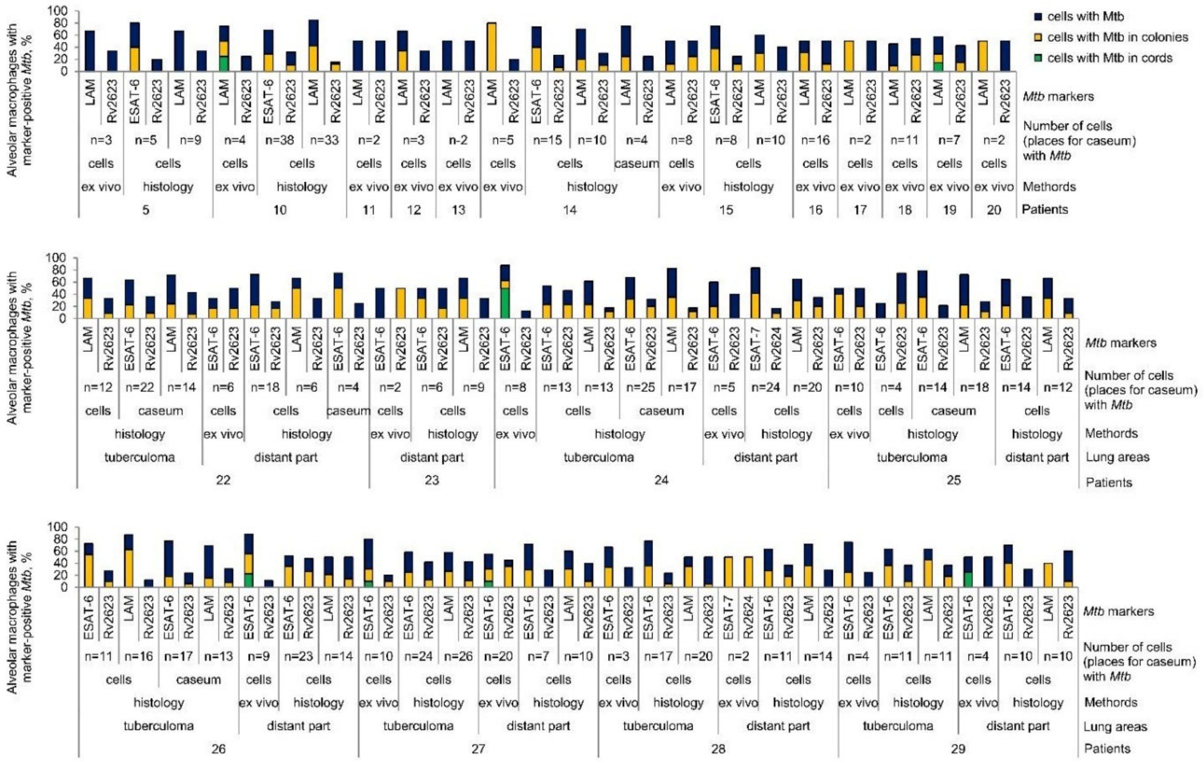

B

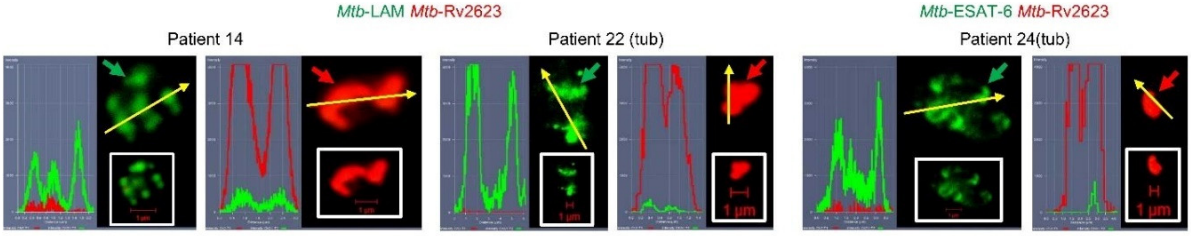

C

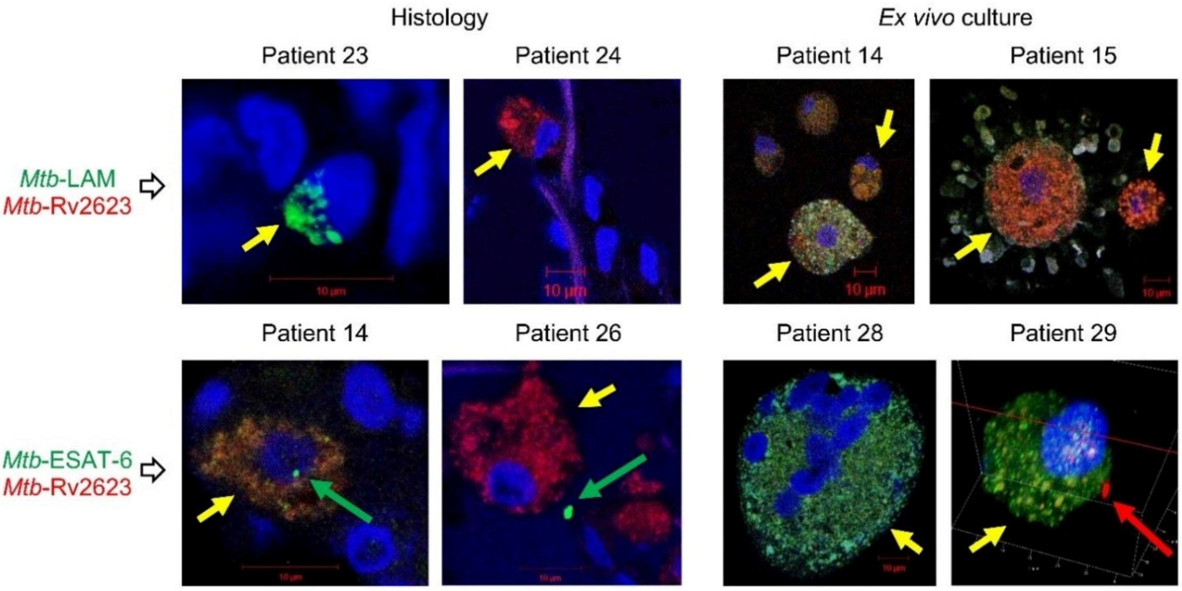

Figure S3. Rv2623-positive *Mtb* present in alveolar macrophages and necrotic caseum in various lung TB lesions of all the patients studied. (**a**) The number of alveolar macrophages or caseous regions with LAM- or ESAT-6- and Rv2623-positive *Mtb* (solitary or as colonies) and with *Mtb* colonies (including those with cording morphology) is expressed as the percentage of the total number of host cells or caseous regions with LAM- or ESAT-6- and Rv2623-positive *Mtb* analyzed simultaneously in them on the histological sections and after *ex vivo* culture for 16-18 hours; (**b**) Representative profile images demonstrate LAM- or ESAT-6-positive *Mtb* (green signal) not expressing Rv2623 protein and, conversely, Rv2623-positive *Mtb* (red signal) not expressing the virulence factors LAM and ESAT-6 in the caseous center of a small granuloma (only for patient 14) and tuberculomas (for other patients) on the histological sections. Yellow arrows point to the areas for constructing profile graphs. Close-ups of the images with the arrow-marked *Mtb* and the scale bar are shown in the lower right part of profile images. The scale bars are 1 μm each; (**c**) The *Mtb* markers stained with antibodies reacting with *Mtb* LAM or ESAT-6 (green signal) and *Mtb* Rv2623 (red signal) often colocalize in the same intracellular vesicles of alveolar macrophages along with or without marker-positive *Mtb* in them on the histological sections and after *ex vivo* cell culture for 16-18 hours. Representative confocal immunofluorescent images are shown. Short yellow arrows point to the alveolar macrophages with intracellular vesicles in them. Long green and red arrows point to ESAT-6- and Rv2623-positive *Mtb*, respectively. The scale bars are 10 μm each.

Figure S4

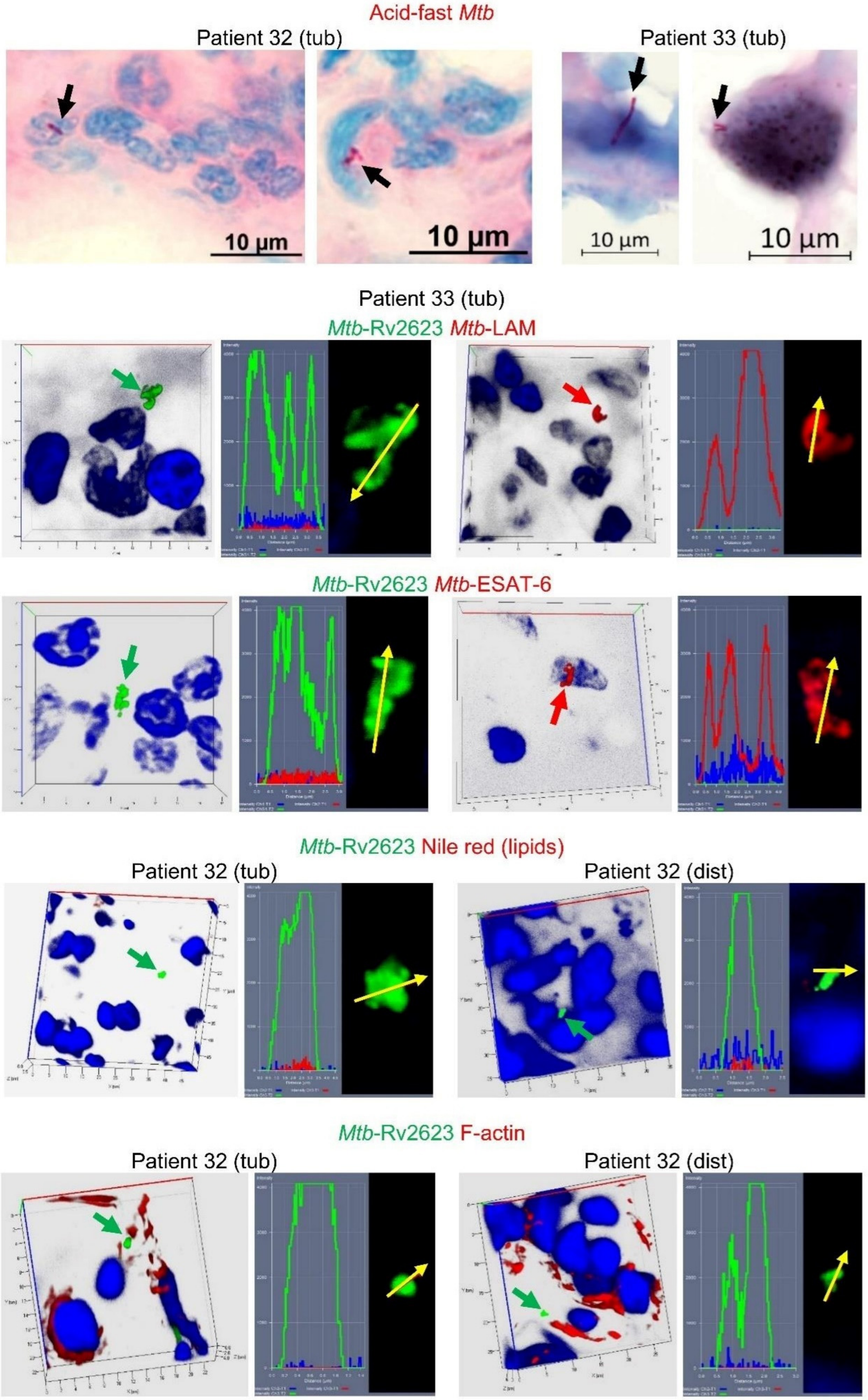

Acid-fast *Mtb*
Patient 32 (tub)
Patient 33 (tub)
10 µm
10 µm
10 µm
10 µm
Patient 33 (tub)
Mtb-Rv2623 *Mtb*-LAM
Mtb-Rv2623 *Mtb*-ESAT-6
Mtb-Rv2623 Nile red (lipids)
Patient 32 (tub)
Patient 32 (dist)
Mtb-Rv2623 F-actin
Patient 32 (tub)
Patient 32 (dist)

Figure S4. Rv2623-positive *Mtb* identified in alveolar macrophages in tuberculoma (tub) walls and tissues distant (dist) from the macro-TB lesions of patients 32 and 33 without any anti-TB treatment before surgery. Representative images of acid-fast *Mtb* after ZN staining are shown in the patients' alveolar macrophages on the histological sections. Representative confocal 3D immunofluorescent images simultaneously stained with appropriate specific antibodies and dyes demonstrate Rv2623-positive *Mtb* (green signal) not expressing the virulence factors LAM and ESAT-6 and, vice versa, LAM- or ESAT-6-positive *Mtb* (red signal) not expressing Rv2623 protein, the absence of intracellular lipophilic inclusions stained by the Nile red dye (red signal), and a lack of colocalization with filamentous actin stained by the Phalloidin dye (red signal) in Rv2623-positive *Mtb* (green signal) in alveolar macrophages on the histological sections. Nuclei are stained by DAPI (blue signal). Green, red, and black arrows point to Rv2623-, LAM- or ESAT-6-positive *Mtb*, and acid-fast *Mtb*, respectively. To the right of the 3D immunofluorescent images: profile images of the arrow-marked *Mtb*. Yellow arrows point to the areas for constructing profile graphs. The scale bars are 10 μm each.

Table S1. Subpopulations (SP) of *Mtb* in alveolar macrophages of patients with pulmonary TB

SP[1]	TB patients[2]										
	1, 11, 12, 21	2	3	4, 23	5	6 (cav), 10	7, 8	9	13	14	15
1	+	+	+	+	+	+	+	+	+	+	+
2	-	+	+	+	-	+	+	+	-	-	-
3	-	-	+	+	+	+	+	+	-	+	+
4	-	-	-	+	-	+	+	+	-	-	-
5	-	-	+	-	-	-	-	-	-	-	-
6	-	-	-	-	-	+	+	-	-	-	-
7	-	-	-	+	-	+	+	+	+	+	-
8	ns	ns	ns	ns	ns	ns	ns	ns	ns	+	+
9	+	+	+	+	+	+	ns	ns	+	+	+
10	-	-	+	+	-	+	ns	ns	-	+	+

<table>
<tr><th rowspan="3">SP[1]</th><th colspan="11">TB patients[2]</th></tr>
<tr><th rowspan="2">16</th><th rowspan="2">17</th><th rowspan="2">18</th><th rowspan="2">19</th><th rowspan="2">20</th><th rowspan="2">22</th><th rowspan="2">26</th><th colspan="2">24, 27</th><th colspan="2">25, 28, 29</th></tr>
<tr><th>tub</th><th>dist</th><th>tub</th><th>dist</th></tr>
<tr><td>1</td><td>+</td><td>+</td><td>+</td><td>+</td><td>+</td><td>+</td><td>+</td><td colspan="2">+</td><td colspan="2">+</td></tr>
<tr><td>2</td><td>-</td><td>+</td><td>+</td><td>-</td><td>+</td><td>+</td><td>-</td><td>+</td><td>-</td><td colspan="2">-</td></tr>
<tr><td>3</td><td>+</td><td>+</td><td>+</td><td>+</td><td>-</td><td>+</td><td>+</td><td colspan="2">+</td><td colspan="2">+</td></tr>
<tr><td>4</td><td>+</td><td>+</td><td>+</td><td>-</td><td>-</td><td>+</td><td>-</td><td>+</td><td>-</td><td colspan="2">-</td></tr>
<tr><td>5</td><td>-</td><td>-</td><td>-</td><td>+</td><td>-</td><td>+</td><td>+</td><td>-</td><td>+</td><td colspan="2">-</td></tr>
<tr><td>6</td><td>-</td><td>-</td><td>+</td><td>-</td><td>-</td><td>-</td><td>-</td><td>+</td><td>-</td><td colspan="2">-</td></tr>
<tr><td>7</td><td>-</td><td>-</td><td>+</td><td>-</td><td>+</td><td>ns</td><td>-</td><td>+</td><td>-</td><td colspan="2">-</td></tr>
<tr><td>8</td><td>+</td><td>ns</td><td>+</td><td>ns</td><td>ns</td><td>ns</td><td>ns</td><td>+</td><td>-</td><td>-</td><td>ns</td></tr>
<tr><td>9</td><td>+</td><td>+</td><td>+</td><td>+</td><td>+</td><td>+</td><td>+</td><td colspan="2">+</td><td colspan="2">+</td></tr>
<tr><td>10</td><td>+</td><td>-</td><td>+</td><td>+</td><td>-</td><td>+</td><td>+</td><td colspan="2">+</td><td colspan="2">+</td></tr>
</table>

(+), is present; (-), is absent; ns, not studied.

[1] Description in Table 2.

[2] Alveolar macrophages were studied in the *ex vivo* cell cultures obtained from the cavity (cav) and tuberculoma (tub) walls and the tissues distant (dist or without labeling) from the macro-TB lesions of the patients' resected lung parts.
